# Cognitive Failure and Alexithymia and Predicting High–Risk Behaviors of Students With Learning Disabilities

**DOI:** 10.5812/ijhrba.16948

**Published:** 2014-06-01

**Authors:** Moslem Abbasi, Mohammad Javad Bagyan, Hamidreza Dehghan

**Affiliations:** 1 Department of Psychology, Faculty of Literature and Human Sciences, Salman Farsi University of Kazerun, Kazerun, IR Iran; 2 Department of Psychology, University of Mohaghegh Ardabili, Ardabil, IR Iran

**Keywords:** Cognition Disorders, Affective Symptoms, Learning Disorders

## Abstract

**Background:**

One of the threatening health issues is prevalence of high-risk behaviors in various groups. Because of rapid social changes, it has been considered as of the most important problems of society by health organizations, administrative laws, and social policymakers.

**Objectives:**

The aim of this study was to determine the role of cognitive failure and alexithymia in predicting high-risk behaviors of students with learning disabilities.

**Patients and Methods:**

This was a correlational research including all 14-16 years old students during 2012-2013 school year in Arak, IR Iran. Eighty students with learning disabilities were sampled by simply random sampling. The data were collected by cognitive failures questionnaire, Toronto alexithymia scale, and high-risk behavior questionnaire.

**Results:**

The results showed that high-risk behaviors had significant positive correlations with difficulty identifying feelings (r = 0.321), difficulty describing feelings (r = 0.336), externally oriented thinking (r = 0.248), distractibility (0.292), memory distortion (r = 0.374), blunders (r = 0.335), and names amnesia (r = 0.275). Multiple regression analysis showed that cognitive failure and alexithymia predicted 32% of the total variance of high-risk behaviors.

**Conclusions:**

These findings demonstrated that cognitive failure and alexithymia had important roles in strengthening and appearance of high-risk behaviors in students with learning disabilities. Therefore, considering those problems, precautionary actions might be necessary.

## 1. Background

Based on fourth edition of the diagnostic and statistical manual of mental disorders, learning disabilities are diagnosed when the progress in standard tests of reading, math and writing are considerably below the expected range, according to age, school grade, and intelligence. On the other hand, learning disabilities in three domains of reading, math and writing, are not normal, despite the intelligence. They must be distinguished of normal problems in cultural progress and school performance related to lack of opportunity, weak teaching, cultural factors, and visual or audition problems (1). The prevalence of learning disabilities has been reported 2-10%. In the past 10 years, the number of students with learning disabilities increased 38% (2). These students, despite the normal intelligence, could not have the desirable academic achievement, problematically continued studying which lead to dropout, causing social, economic, cultural, mental, and emotional deficiencies and finally delinquency (2). High-risk behaviors are psychological variables that may be resulted from learning disabilities in students. They are known as behaviors that increase the risk of physical, psychological, and social consequences in a person. These behaviors are strongly correlated with adolescents and follow a covariational pattern. Researchers, by presenting the problem behavior syndrome, introduced high-risk behaviors as smoking, substance and alcohol abuse, dangerous driving, and early sexual activity (3). A longitude study showed that of 22 children, diagnosed as learning-disabled, about 27% of 17-18 years old ones perpetrated high-risk behaviors and were delinquency cases (4). A longitude study illustrated that of 8-12 years old students with learning disabilities, about 65% perpetrated high-risk behaviors and alcohol abuse was the most observed action (5).

Cognitive failure is one of the variables that children with learning disabilities are subject to. Cognitive failures are known as errors or faults of a person in performance of tasks, which they can naturally perform. On the other words, cognitive failure is a multidimensional construct that include the errors in object formation, schemas activation, and action triggering (6). Due to interference with daily activities, cognitive failures can lead to basic problems. Sometimes, compensation of these errors needs a long time, and their occurrence may lead to serious difficulties and even death (7). Most of the researchers have agreed that cognitive failures, including distractibility, memory distortion, blunders, and amnesia, are closely related to detection of high-risk behaviors in adolescents (8). Furthermore, there has been a significant positive correlation between cognitive failure and tendency to delinquent and high-risk behaviors (9). Literature shows the relationship between cognitive failure and various components of high-risk behaviors such as alcohol consumption, substance abuse, dangerous driving events, and high-risk sexes (10).

Alexithymia is a subclinical condition characterized by difficulties in identifying and describing one’s own emotional state. For example, individuals with alexithymia might know that they are experiencing an emotion, but be unaware whether that emotion is sadness, anger or fear. Although the incidence of alexithymia in the typical population is estimated as 10%, elevated levels of alexithymia have been seen in a number of disorders such as anorexia nervosa, substance abuse, and post-traumatic stress (11). Alexithymia is another problem in students with learning disabilities. It is defined as problem in emotional self-regulation and disability of cognitive processing of emotional information (12). Individuals with Alexithymia have serious problems in recognition and describing personal emotions symptoms, limiting expression of feedbacks, affects, tendencies, and drives. Students with difficulties in reading and writing, compared to their normal peers, had alexithymia, weakness in social interactions, depression, negative effects, and bulling behaviors with higher degrees (13). Antisocial, aggressive, and high-risk behaviors in students with learning disabilities are significantly more than normal students (14). Children with learning disabilities have problems in emotional stability, and also more severe reactions encountering emotional situations, preparing them to perpetrate high-risk behaviors such as dangerous diving (15).

Children with learning disabilities that have emotional and cognitive deficiencies are more tended to high-risk behaviors (16). Totally, considering the high prevalence of this disorder in students, the role of emotion and cognition as the key factors for success, health enhancement, and decrease of psychological difficulties and high-risk behaviors must be considered. There are scarce researches in this context; thus, use of this research for psychopathology of learning disabilities should be considered.

## 2. Objectives

The aim of this study was to determine the roles of cognitive failure and alexithymia in predicting high-risk behaviors of students with learning disabilities.

## 3. Patients and Methods

The research was descriptive and correlational. Cognitive failure and alexithymia were assumed as the predictor variables and high-risk behaviors as criteria variables. The community of study included all 14-16 years old students during 2012-2013 in Arak city, Iran. Firstly, all learning-disabled students in this age range were identified in exceptional children schools; then, 80 of them were sampled by simple random sampling. Considering the fact that in correlational studies the minimum number of samples must be 30 to 50, and for increasing the external validity of research, our sample size was selected 80 (17).

### 3.1. High-Risk Behavior Questionnaire

In this study, high-risk behavior questionnaire was used to determine the prevalence and identify the maintaining and incentive factors of high-risk behaviors (3). It included 38 items and the answers were based on a five-point Likert-type scale (ranging from absolutely agree to absolutely disagree). This scale has seven components (tendency to substance abuse, alcohol, violence, sexual relation, sexually offensive behavior, and dangerous driving). Psychometric properties of the high-risk behavior questionnaire in the standardized version by Mohamadi were hopeful to distinguish the groups. Its construct validity was reported 0.77 by Nariman and (14) and its reliability was reported as Cronbach's alpha = 0.67.

### 3.2. Cognitive Failures Questionnaire

Cognitive failures were measured by cognitive failures questionnaire, comprised of 24 items (18). Each item was on a five-point Likert-type scale (ranging from never to always) with four subscales of distractibility (nine items), memory distortion (seven items), blunders (seven items), and names amnesia (two items). The Cronbach's alpha coefficients for all the questionnaire items were reported 0.84 in two researches. in one research, it was reported 0.79, 0.64, 0.66, and 0.62 for all the subscales, respectively (15, 19). Abolghasemi, in a preliminary study regarding validation of the cognitive failures questionnaire, obtained 0.89 for internal consistency and 0.77 for retest reliability coefficients after one month for an Iranian sample (100 individuals) (20).

### 3.3. Toronto Alexithymia Scale (TAS-20)

The children and adolescent form of Toronto alexithymia scale has been derived of adults form Alexithymia Scale, and developed by Rieffe, Oosterveld and Terwogt (15), that has 20 items. Each item is on a three-point Likert-type scales (absolutely, somewhat, and not at all). Three factors including difficulty identifying feelings, difficulty describing feelings, and externally oriented (objective) thinking style, were evaluated by TAS-20. The Cronbach's alpha of this scale was obtained 0.84. The correlation coefficients of subscales with SCL–90-R were reported 0.48-0.70 (21), and the correlations of alexithymia scale with mental annoyance (R = 0.34) and mental inadvertency (R = -0.20) were significant (P < 0.01) (22). Besharat reported the validity of three subscales of difficulty identifying feelings, difficulty describing feelings, and externally oriented thinking, 0.85, 0.82, and 0.72, respectively (22), suggesting an appropriate validity for Iranian culture.

### 3.4. Procedure

After confirmation of the Arak city Office of Education and earning the subjects’ satisfactions, the learning-disabled students were identified and the aim of research was explained for them. Cognitive failure and alexithymia were assumed as the predictor variables and high-risk behaviors as criteria variables. Firstly, all the learning-disabled students in this age range were identified in exceptional children schools, and 80 of them were sampled by simple random sampling. The questionnaires were provided and the students were asked to fill them carefully and completely and select the desirable responses according to their characteristics. The data was individually collected from each school and analyzed by multiple regression analysis and Pearson correlation coefficient. In addition, data confidentiality and optional participation were among the moral points in our research.

## 4. Results

Means and standard deviations of our sample were as follows: age 14.36 (3.26), difficulty identifying feelings 21.48 (3.45), difficulty describing feelings 17.36 (3.45), externally oriented thinking 22.77 (4.16), total alexithymia scale 61.61 (11.01), distractibility 27.25 (5.12), memory distortion 22.45 (3.36), blunders 23.26 (4.55), names amnesia 8.36 (1.2), total cognitive failures 81.32 (14.23), tendencies to substance abuse 22.32 (3.87), alcohol abuse 21.18 (3.23), cigarette smoking 18.36 (2.45), violence 19.47 (2.36), sexual relations and behaviors 12.63 (2.10), sexually offensive behaviors 11.57 (2), dangerous driving 18.25 (3.18), and total high-risk behaviors 120.81 (19.19).

The literacy level of fathers and mothers were accordingly 66% and 71% below high school diploma, 26% and 26% high school diploma, 5% and 4% associate degree, and 3% and 3% BA degree. Among participants, 43.9% were first child, 29.78% second, and 26.32% third and above. Occupational status of fathers and mothers were respectively 20% and 11.36% employee; 80% of fathers were self-employed and 88.64% of mothers were housewives.

As Table 1 shows, difficulty identifying feelings (r = 0.321), difficulty describing feelings (r = 0.336), externally oriented thinking (r = 0.248), distractibility (r = 0.292), memory distortion (r = 0.374), blunders (r = 0.335), and names amnesia (r = 0.275) had significant positive correlations with high-risk behaviors (P < 0.001) (Tables 1 and 2). This means that emotional and cognitive failures can create further tendency to high-risk behaviors. To determine the effect of any variable, difficulty identifying feelings, difficulty describing feelings, externally oriented thinking, distractibility, memory distortion, blunders, and names amnesia were analyzed as predictor variables and high-risk behaviors as the criteria variables, by multiple regression analysis in enter way.

**Table 1.** Correlation Coefficients Among Cognitive Failures and Alexithymia Components and High Risk Behavior

	1	2	3	4	5	6	7	8
**Difficulty identifying feelings**	1							
**Difficulty describing feelings**	0.304 ^a^	1						
**Externally oriented thinking**	0.612 ^a^	0.303 ^a^	1					
**Distractibility**	0.156 ^a^	0.131 ^b^	0.285 ^a^	1				
**Memory distortion**	0.216 ^a^	0.169 ^b^	0.314 ^a^	0.501 ^a^	1			
**Blunders**	0.206 ^a^	0.203 ^a^	-0.200 ^a^	0.536 ^a^	0.338 ^a^	1		
**Names amnesia**	0.163 ^b^	-0.010	-0.005	0.483 ^a^	0.295 ^a^	0.315 ^a^	1	
**High-risk behaviors**	0.321 ^a^	0.336 ^a^	0.248 ^a^	0.292 ^a^	0.374 ^a^	0.335 ^a^	0.275 ^a^	1

^a^ Correlations are significant at P < 0.05.

^b^ Correlations are significant at P < 0.001.

**Table 2.** The Results of the ANOVA for eaningful Assessment of the Entire Model ^a^

	SS	DF	MS	F	P Value
**Model**				9.217	0.001
Regression	11209.204	4	1401.151		
Residual	23259.296	76	152.022		
Total	34468.500	80	152.022		

^a^ Based on the table above, and the obtained value of F (9.217) independent variables explanatory power has been strong and well able to explain the changes and the variance in the dependent variable.

As Table 3 shows, the observed F is significant and results showed that 32% of variances of high-risk behaviors were explained by the variables of difficulty identifying feelings, difficulty describing feelings, externally oriented thinking, distractibility, memory distortion, blunders, and names amnesia. According to the standard coefficients (beta) values, difficulty identifying feelings with β = 0.262, difficulty describing feelings with β = 0.112, externally oriented thinking with β = 0.145, distractibility with β = 0.187, memory distortion with β = 0.189, blunders with β = 0.169, and names amnesia with β = 0.341 were the strongest predictor variables of high-risk behaviors (Table 3).

**Table 3.** Multiple Regression Analysis Results of Cognitive Failures and Alexithymia Components and High Risk Behavior ^a^

Predictor Variable	R	R^2^	RS	Nonstandard Coefficient		Standard Coefficient (Beta)	t	P Value
				SE	B			
**Constant**	-	-	-	11.605	110.353	-	9.509	0.000
**Difficulty identifying feelings**	0.358	0.128	0.117	2.187	7.834	0.262	3.582	0.000 ^a^
**Difficulty describing feelings**	0.375	0.141	0.125	1.906	2.813	0.112	1.476	0.006 ^a^
**Externally oriented thinking**	0.388	0.151	0.129	2.079	2.582	0.145	1.539	0.003 ^a^
**Distractibility**	0.459	0.211	0.186	0.242	0.380	0.187	2.237	0.000 ^a^
**Memory distortion**	0.469	0.220	0.190	0.370	-0.791	-0.189	-2.137	0.034 ^b^
**Blunders**	0.492	0.242	0.207	0.421	0.846	0.169	2.011	0.046 ^a^
**Names amnesia**	0.570	0.325	0.290	0.111	0.483	0.341	4.345	0.000 ^a^

^a^ P < 0.001.

^b^ P < 0.05.

## 5. Discussion

The purpose of this study was to determine the role of cognitive failures and alexithymia for predicting high-risk behaviors in students with learning disabilities. Findings showed significant relation between cognitive failures and high-risk behaviors. In other words, students with high cognitive failures experienced higher risk behaviors. This was accordant with other researches (3, 6, 7, 13, 14). To explain these results, it can be mentioned that metacognition or knowledge of cognition can be a strong predictor of academic achievement in school. In fact, rather than being a subject matter, cognition is a method of thinking based on the ability of understanding, presenting the problem states, explaining the fundamental concepts of the problem, and organizing and classifying the required information. In other words. On the other hand, that is defined knowledge of an individual about his own information and circumstance of his learning, as a domain of executive functions like attention, monitoring, control, planning and error discernment (23). This information is often subjective and can affect the behavior (13). Students with learning disabilities, due to of unawareness on their emotions and cognitions and inability to execute functions, are more likely to have stress, depression, anxiety and aggression (6). Stress and its negative effects due to dwindling the cognitive abilities, prevent accurate processing of information and lead to distractibility, memory distortion, and cognitive fault (24). These students due to their cognitive failures have lower attention and weaker performance of tasks which need attention, encoding and maintaining matters, compared to their peers. In other words, an individual with cognitive failures is always in a harmful uncertainty. In such situation, the person arouses for releasing this uncertainty. He/she has problems in preservation of matters, feels undesirability and worthlessness that can lead to loneliness, and has self-blame, depression, and unplanned and impulsive behaviors (25). Therefore, cognition, beliefs, and thought dimensions have important portions in resistance and negative interpretations of internal experiences, such as those that lead to anxiety, anger and aggression (3). Another explanation is that parenting methods that include rejection, emotional deprivation and high control, may cause development of undesirable cognitive schemas which lead to a type of worthlessness and isolationism in child (18). In contrast, supervision and sincerity of parents and desirable emotional relation can develop a secure attachment in child, causing a cognitive schema with sincerity, self-esteem and increased positive affects (9).

The findings suggested a significant relation between alexithymia and high-risk behaviors. Therefore, children with higher emotional deficiencies experiences higher-risk behaviors. This is accordant with other researches (3, 6, 13, 14, 17). To explain these findings, we can say that alexithymia is a cognitive-emotional characteristic and a person with alexithymia is unable to understand his own emotions and regulate them. In fact, when the emotional information cannot be perceived and evaluated in cognitive processing, individuals emotionally and cognitively experiencen disturbance and agitation. This inability can disturb their organization of cognitions and emotions (6). These children, usually because of the lack of sincere relationships and enough stable emotional connection to their parents, unawareness of their parents’ emotions, and inability in cognitive processing of their feelings, are unable to identify, perceive, and describe their own emotions, and have limited capability in adapting to stressing situations (5). One of the methods of controlling stress, especially about emotions, is discharging them. When a person cannot verbally express feelings, the psychological part of emotions expression and mental disturbance such as depression, anxiety and aggression gets activated. Individuals that can identify their feelings and express their various states of emotions, can well encounter life problems and are more successful in adjusting with others and environment, leading to their further mental health (14). Nowadays, the state of child-parent relationship is of the most basic research backgrounds. The children research findings clearly suggested that child is not a passive creature and is influenced by familial traits, parenting styles, couples relationships, and quality of expression, explanation and management of emotions in parents. A research showed that 38-78% of children with emotional deficiencies had learning disabilities (21). One probable reason for emotional problems of students with learning disabilities is that they have deficiencies in the emotional cognition domain. They wrongly interpret social symbols and may invert them. These children have problems in distinguishing tag indications, situational indicators, and encoding the nonverbal emotional signs, leading to high levels of aggression and disturbed behaviors in interpersonal relationships (26). Multiple regression analysis showed that components of alexithymia and cognitive failures significantly explained high-risk behaviors. These variables predicted 32% of high-risk behaviors variances (P < 0.001). Difficulty identifying feelings component with β = 0.262, distractibility with β = 0.187, and names amnesia with β = 0.341 were the strongest predictors of high-risk behaviors. To explain these findings, we can say that individuals with low scores in alexithymia, have more mental abilities to process social information. This ability can help them to have a better perception of negative and harmful consequences of substance abuse, cigarette smoking, alcohol consumption, and other high-risk behaviors, and thus, be more successful to resist against mental and social stresses pushing them to high-risk behaviors (5). In fact, they can enhance their social relationships by accurate formulation and process of information and distinguish having right or wrong behaviors in different situations. In contrast, students with learning disabilities, due to the lack of self-regulation and accurate formulation of information, lack social and interpersonal skills. Therefore, they encounter more problems in life and feel a type of disgust from society, mostly leading to isolation from community; instead of performing internal planned behaviors, they tend to perverse behaviors. On the other hand, depressed mode in these individuals, based on self-treatment model, is as a device to transform the stressing factors (i.e. anxiety, depression, anger, aggression). These students use the physiological and psychological properties of materials to regulate and adjust their negative emotions and obtain emotional stability. In fact, they relieve themselves by misusing their preferred substance and consequently bear their emotional situations (27).

People with learning disabilities and mental health problems also have an extraordinary range of physical disorders (including epilepsy) which makes their presentation even more complex. For some people who present challenging behaviors, physical and mental health issues are intricately linked together and it can often be difficult to tease out whether the presentation is due to an underlying organic (physical) condition. In many of these complex presentations, continuous nursing observation, physical investigations, and medical and psychiatric expertise may be needed within an in-patient setting for accurate diagnosis and effective treatment (28).

Finally, in this study, the small sample size and impossibility of comparing the study variables to 12-16 years old learning-disabled female students because of inaccessibility to them in our community schools and centers were of the most important limitations. We hope that in future researches this comparison can be performed. It is suggested that managers of these centers help these students to accurately express their emotions by their effective supports and facilitate and enhance emotional, cognitive and mental adjustments of these children.
